# Editorial: Metabolic Shifting: Nutrition, Exercise, and Timing

**DOI:** 10.3389/fnut.2020.592863

**Published:** 2020-12-10

**Authors:** Tanya Zilberter, Antonio Paoli

**Affiliations:** ^1^Independent Researcher, Marseille, France; ^2^Department of Biomedical Sciences, School of Medicine and Surgery, University of Padua, Padua, Italy

**Keywords:** metabolic adaptations, metabolic clock, shift work, time-restricted feeding, time-restricted eating, fasting, ramadan fasting, ketogenic diet

From the teleological standpoint, metabolic shifting evolved due to the periodic nature of the environment, circadian cycles being one of the conditions the life on Earth confronts. Metabolic shifting occurs on a regular basis due to the demand-availability interplay of energy resources, expenditure, and depots, all of which have periodic nature due to the environment's circadian rhythmicity. In modern societies, however, there are discrepancies between the evolutionary acquired metabolic setup and our current dysrhythmic, artificial environment. Thus, the so-called “diseases of civilization” are thought to be due to the changes in the environment that are too fast to cause adequate adaptations in our genome. On the other hand, an inherent fundamental feature of metabolism at large is flexibility. Out of the many diverse manifestations of metabolic flexibility, this Research Topic addresses the triad: “*Timing, Nutrition, and Exercise*” for the apparent reason—they are areas where they depend on each other and intertwine.

## Nutrition

Just a few days of nutritive imbalance, e.g., on the obesogenic diet (high in both fat and sugar), resulted in weight gain and loss of metabolic control resulting in impaired hypothalamic glucose sensing and hippocampal insulin signaling. These pathologies, in turn, led to obesity, metabolic syndrome, and the hippocampal-dependent memory function (Garcia-Serrano and Duarte). Nutrients entrain peripheral tissue clocks via the molecular clocks. Carbohydrate intake-induced insulin secretion activates clock genes. Glucose tolerance is higher at the onset of the daytime than in the evening. Fuel selection in a skeletal muscle during exercise depends on the time of day. At night, high-fat meal intake caused a more noticeable increase in triacylglycerol levels than in the daytime. Some nutrients like selenium or the flavonoid nobiletin, to name just two, are capable of enhancing circadian rhythms, e.g., via the clock genes (Geng et al.), which may have significant practical applications in cases of environmental or pathological dysrhythmias (Potter and Wood).

The master clock in the suprachiasmatic nucleus is the lead light-dark pacemaker of the body. Still, the bodily structures insensitive to light nevertheless participate in metabolic alignments via their responsiveness to feeding and fasting. Shift work, feeding during the inactive phase, permanent food availability (the *ad libitum* experimental protocol), overly palatable foods—all adversely influence the peripheral molecular clocks and metabolic homeostasis (Pickel and Sung).

An essential part of timing as a whole is feeding/eating regimens restricting food availability to a certain period of the active phase of the day. For instance, intermittent fasting and time-restricted feeding are mentioned in most of the participating papers (Bae et al.; BaHammam and Almeneessier; Garcia-Serrano and Duarte; Norgren et al.; Potter and Wood; Pickel and Sung; Wang et al.).

Restricting feeding prevents diet-induced obesity without changing energy intake than the *ad libitum* protocol while improving the essential metabolic biomarkers. On the other hand, unrestrictive obesogenic diets adversely impact circadian rhythms and metabolic parameters (Bae et al.). The undernutrition approach to disease prevention and treatment (e.g., fasting) is known since ancient times. It is in use in our days as an antidote to obesogenic overnutrition and environments in the form of complete or intermittent fasting or time-restrictive eating/feeding protocols including.

From the physiological viewpoint, Ramadan fasting is a time-restrictive eating regiment, which does not seem to disturb the circadian rhythms and, as time-restrictive eating/feeding or intermittent fasting generally, has beneficial effects, e.g., on lipid, glycemic, and inflammatory biomarkers (BaHammam and Almeneessier).

## Exercise

Homeostatic balance is based on constant monitoring of energy intake vs. energy expenditure. Exercise is a potent entrainment factor for central as well as peripheral clocks, including such in muscle, where fuel selection depends on the time of day as are postprandial effects of nutrients. It may prevent autophagic or apoptotic dysfunctions. Aerobic exercise training improves risk factors of metabolic syndrome such as glucose intolerance, hyperlipidemia, high blood pressure, low high-density lipoprotein content, and visceral obesity. Improved aerobic fitness increases neurogenesis, enhances memory, and may prevent brain atrophy. Adequate-intensity exercise reduced prenatal depression and shortened early labor stages. Physical activity enhances cognitive functions in adolescents, and young adults attenuate or prevent such autophagic or apoptotic dysfunctions (Andreotti et al.).

## Timing

There is the two-way communication between metabolic signals and circadian processes such as rhythmicity of feeding, gene expression, enzymatic, and metabolic pathways. The authors of the Topic address the consequences of these communications for a diverse array of interactions, including the sleep-wake cycle, body temperature, hormonal processes, dietary composition, human social, and occupational behaviors. The environmental cycles (diurnal rhythms, temperature variations, seasonal food opportunities) interact with the endogenous ones at the cellular and organismic levels. Such a complex networking mechanism is not immune to the rhythmic desynchronization resulting in a plethora of metabolic disarrays (Bae et al.).

The circadian clock drives physiological functions controlling energy homeostasis. In their turn, parameters of energy intake and expenditure influence the circadian clock, e.g., the expression of the clock genes. Food intake, fuel selection, and exercise capacity depend on the circadian clock and are time-of-day dependent (Aoyama and Shibata). Feeding rhythms and dietary compositions are sensitive to many aspects of feeding behaviors and thus are capable of regulating the transcriptional, enzymatic, metabolic, and microbiome processes (Bae et al.).

Other essential confounders impacting the metabolic markers are the light exposure and sleep-wakefulness timing and energy expenditure fluctuations. Restriction of food availability exclusively to the active phase prevents weight gain and metabolic syndrome independently from caloric restriction while eating during the inactive period (nighttime eating in humans) increases the likelihood of obesity (BaHammam and Almeneessier). The timing of food intake may contribute to metabolic shifting, for instance, to ketosis, depending on the macronutrient ratio and calorie intake (Norgren et al.). Diet composition and timing affect the circadian clock and are powerful enough to potentially help shift workers adapt to their schedules (Potter and Wood).

Summing up, this Topic offers the latest experimental and analytical works concerning characteristics of environmental cyclicity, its effects on metabolic adaptations and maladaptations.

## Author Contributions

All authors listed have made a substantial, direct and intellectual contribution to the work, and approved it for publication.

## Conflict of Interest

The authors declare that the research was conducted in the absence of any commercial or financial relationships that could be construed as a potential conflict of interest.

